# Information Hiding in the DICOM Message Service and Upper Layer Service with Entropy-Based Detection

**DOI:** 10.3390/e24020176

**Published:** 2022-01-25

**Authors:** Aleksandra Mileva, Aleksandar Velinov, Vesna Dimitrova, Luca Caviglione, Steffen Wendzel

**Affiliations:** 1 Faculty of Computer Science, “Goce Delcev” University, 2000 Shtip, North Macedonia; aleksandar.velinov@ugd.edu.mk; 2 Faculty of Computer Science and Engineering, Ss. Cyril and Methodius University, 1000 Skopje, North Macedonia; vesna.dimitrova@finki.ukim.mk; 3 Institute of Applied Mathematics and Information Technologies, 16149 Genova, Italy; luca.caviglione@ge.imati.cnr.it; 4 Center for Technology and Transfer, Worms University of Applied Sciences, 67549 Worms, Germany; wendzel@hs-worms.de

**Keywords:** network steganography, medical informatics, HIS, PACS, CT scanners, MRI scanners, ultrasound

## Abstract

The DICOM (*Digital Imaging and COmmunication in Medicine*) standard provides a framework for a diagnostically-accurate representation, processing, transfer, storage and display of medical imaging data. Information hiding in DICOM is currently limited to the application of digital media steganography and watermarking techniques on the media parts of DICOM files, as well as text steganographic techniques for embedding information in metadata of DICOM files. To improve the overall security of the DICOM standard, we investigate its susceptibility to network steganographic techniques. To this aim, we develop several network covert channels that can be created by using a specific transport mechanism – the DICOM Message Service and Upper Layer Service. The bandwidth, undetectability and robustness of the proposed covert channels are evaluated, and potential countermeasures are suggested. Moreover, a detection mechanism leveraging entropy-based metrics is introduced and its performance has been assessed.

## 1. Introduction

**Figure 1:**
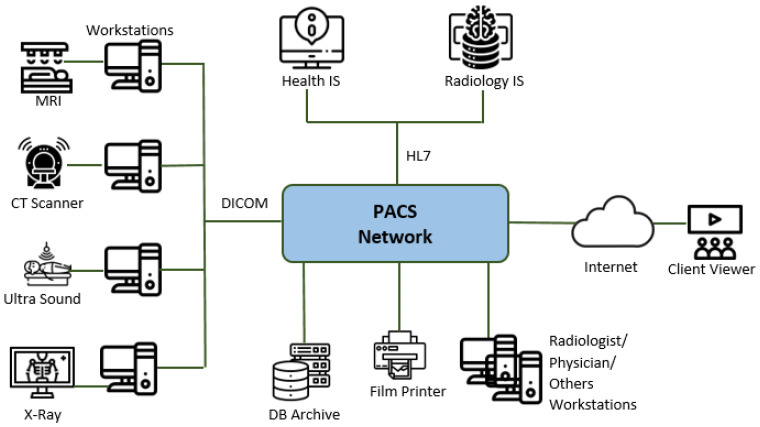
Reference design for a PACS network infrastructure.

Contemporary hospitals implement electronic medical records and large information systems (IS), integrated by computer networks and software applications. There are different types of information systems. First, there is a hospital information system (HIS), which is a computerized management system for handling and supporting clinical operations, medical patient care activities, hospital’s business and administrative functions, etc. [1]. Also, there is a radiology information system (RIS) for the radiology department, which manages patient-related information (e.g., medical, patient demographics, and billing information), and examination-related information (e.g., patient arrival documentation, procedural descriptions, diagnostic reporting). The various IS exchange information by using the HL7 standard (https://www.hl7.org, accessed on 5 December 2021). In this scenario, an important part is the Picture Archiving and Communication Systems or the PACS infrastructure responsible of integrating the *modalities*. The latter, according to the standard, are medical image acquisition devices (such as CT scanners, MRI scanners, ultrasound, etc.); *archives* for storing medical images; *workstations* where radiologists and other practitioners view, re-process, and interpret the images; and *printers* for printing images [2] (Figure 1).

The glue that holds all these components together is the *Digital Imaging and COmmunication in Medicine* (DICOM) standard (https://www.dicomstandard.org/current/, accessed on 5 December 2021)), established in 1985 by the American College of Radiology (ACR) and the National Electrical Manufacturers Association (NEMA). DICOM provides file formats for representing medical images and related data (e.g., patient information, modality and imaging procedure information, image information). Moreover, it provides a framework, services and tools for processing, storing, displaying and transferring medical imaging data between all the components and different vendors’ equipment. Each PACS device implements only the subset of the DICOM standard required for the particular task (presented in the *DICOM Conformance Statement* of the specific device or its software).

While the healthcare digitization brings many advantages, like shortened time of everyday hospital activities, better usability, etc., on the negative side, it turns the medical institutions into a new and valuable target of cybercriminals. We are witnessing many recent attacks on medical institutions that involved data breaches (https://www.nytimes.com/2015/02/05/business/hackers-breached-data-of-millions-insurer-says.html, accessed on 5 December 2021), ransomware (https://www.nbcnews.com/tech/security/cyberattack-hits-major-u-s-hospital-system-n1241254, accessed on 5 December 2021), insider threats (https://archives.fbi.gov/archives/dallas/press-releases/2011/dl031811.htm, accessed on 5 December 2021) and DDOS attacks (https://thethreatreport.com/story-behind-the-ddos-attack-vs-boston-children-hospital/, accessed on 5 December 2021)—just to mention a few. The attackers can even add or remove evidence of medical conditions from medical scans, with the goal to change the patient’s diagnosis and its outcome [3]. Duggal [4] demonstrated different attack scenarios on medical devices and healthcare infrastructure, such as gaining patient information, fingerprinting architecture, examining and changing diagnosis, gaining access to non-prescribed drugs, changing medications, DoS attacks on medical equipment, etc.

As shown in our previous work, DICOM files can also be used for steganographic purposes [5]. Steganography deals with the concealment of secret information, including its storage and transfer. Several digital artifacts have been investigated regarding both, steganography application and its detection, including digital text, digital images, videos and audio files, filesystems, cyber-physical systems, and networks. By using steganography, it is possible to create parasitic communication paths, which are often defined as *covert channels*, see, e.g., Refs. [6,7,8] for an overview on the topic. Specifically, data can be hidden within a suitable carrier (e.g., part of a protocol data unit that is not used or optional) to allow remote endpoints to covertly exchange information. There are many envisioned scenarios for hiding data in DICOM files and traffic [5]. On the illicit side, they include the creation of abusive communication paths to orchestrate infected devices, drop malicious payloads to spread infections, perform scanning or data gathering campaigns, create channels for moving data among different devices that are supposed to be isolated, implement privacy-leaking and data exfiltration campaigns, just to mention the most popular use cases. On the contrary, legitimate applications include the detection, limitation or prevention of threats or malware endowed with information-hiding capabilities, “augmentation” of the various DICOM dataflows and entities with secret data in order to void machine-learning-capable attacks, as well as the design of suitable algorithms for the prevention of malicious tampering and alteration of DICOM images.

In the last years, it became common practice to categorize steganographic methods (including covert channels) using so-called *hiding patterns*, which were introduced in [9], while their latest version is present in [10]. In the rest of this paper, we will denote the suitable pattern by reporting it between parenthesis, i.e., by using the notation borrowed from [10].

To summarize, in this paper we showcase how to apply steganographic techniques on the DICOM Message Service and Upper Layer Service with the goal of creating covert channels. The main contributions of this work can be summarized as follows:

- It shows the existence of ten new covert channels using the DICOM standard
- It categorizes the determined covert channels using the latest version of the hiding patterns taxonomy [10];
- It showcases an entropy-based detection methodology for real scenarios and known covert channels;
- It performs and experimental campaign to show how irregularities in Message ID can reveal when covert traffic is present.

The rest of the paper is structured as follows. Section 2 reviews the related work, while Section 3 describes the basic of DICOM. Section 4 describes the newly suggested covert channels. We cover metrics and potential countermeasures in Section 5. An entropy-based detection method as well as its performance evaluation is shown in Section 6. The concluding remarks of this paper are made in Section 7.

## 2. Related Work

According to [3], Shodan.io allows to reach 1849 DICOM servers and 842 PACS servers, which have been exposed to the Internet via web interfaces. Despite the intentional nature, the volume of leakage in terms of DICOM images and personal data represents a real-world hazard. In more detail, poor security requirements, (e.g., default accounts, cross-site scripting, unencrypted traffic, vulnerabilities in the web servers) characterizing various DICOM/PACS deployments can be exploited to view and modify 3D DICOM images [11]. Moreover, the impact in terms of insecurity is also largely due to open source or small-fee PACS that are often used in small health care institutions or practices [11]. Therefore, DICOM assets are expected to become a valuable target for attackers in the near future [12]. Fraud attempts are another important drive for bypassing the security of DICOM contents. As a paradigmatic example, Mirsky et al. [3] recently demonstrated how a malicious threat can remove evidences of medical conditions from volumetric medical scans. Specifically, by using a 3D conditional GAN, they showcased how to deceive radiologists and state-of-the-art AI screening tools.

The increasing diffusion of ransomware attacks against carefully-selected targets can find a highly profitable ecosystem in PACS/DICOM deployments (see, e.g., [13]). Yet, the complexity of the standard, the heterogeneity of various deployments, and the widespread digitization of medical contents are effective drives for exploiting DICOM contents for information hiding and steganographic purposes. In fact, as shown in our preliminary work [5], which is extended by this paper, DICOM artifacts can be used to encode secret data via text steganography (e.g., by hiding a secret message through the manipulation of whitespaces), thus enabling a wide array of privacy-breaking attacks or mass profiling attempts. Taking into account that DICOM images are a combination of normal images with a lot of metadata, any steganographic techniques that can be applied to normal images can be applied to DICOM images, too. Several such techniques exist, see, e.g., the surveys [14,15]. For example, Sahu and Swain [16] use the difference between a pair of consecutive image’ pixels and two embedding/extracting functions (based on an adaptive range table) to hide the data. For medical images, one can find it useful to apply the reversible data hiding approaches which completely recover the cover image together with the secret data, such as the two methods presented in [17]. For example, in one of the methods, four identical images are created from the cover image, and then, by using *n*-rightmost bit replacement and modified pixel value differencing, the secret bits are embedded in all four images.

However, the importance of investigating the applicability of steganographic techniques on DICOM content is not only limited to understanding its offensive potentials. In fact, *The Health Insurance Portability and Accountability Act* (https://www.cdc.gov/phlp/publications/topic/hipaa.html, accessed on 5 December 2021) requires the adoption of security measures in order to protect sensitive patient information (including metadata). In this vein, watermarking of medical images is an important topic to fully assess the security of DICOM and guarantee its deployment for carrier-grade utilization (see, e.g., [18,19] and references therein). As a possible example, Mantos and Maglogiannis [20] developed an algorithm for hiding sensitive patient data to ensure their integrity and recognize manipulations of critical part of the image. To this aim, they used a variant of the Least Significant Bit (LSB) encoding method to embed 2 bit of secret data per pixels and “mark” regions of interest to improve their robustness against tampering and manipulation attempts.

## 3. DICOM Basics

DICOM provides an object-oriented abstract data models named *Information Object Definitions (IODs)* (PS3.3), that represent any DICOM-related entity, like patients, studies, images, clinical trials, etc. IOD can act as a single class, called *Normalized IOD* (e.g., Print Job IOD), or as a collection of several classes, called *Composite* IODs (e.g., CT Image IOD). Appropriate data objects consist of a number of attributes, such as patient name, ID, gender, age for patients, or a data stream of the pixels that comprise a concrete image. The attributes are formatted according to 34 *Value Representation (VR)* types, and are listed in the DICOM *Data Dictionary* (PS3.6).

Each DICOM file contains a header and a single structured set of attributes (*data elements*) named as a *data set*. In the data set, the attributes are uniquely identified and ordered (in ascending order) by an ordered pair of 16-bit unsigned integers (Group Number, Element Number), named as *data element tag*. Their format, length and value are defined in the VR field, Value Length field and Value field. The attributes can have only values with sizes of even number of bytes.

DICOM provides an order to all these data elements, by introducing the *Patient-Study-Series-Image* hierarchy [2]. This hierarchy means that one patient may have multiple studies (e.g., MR or CT exams), each study may include one or more image series (e.g., CT or ultrasound), and each series has one or more images.

Particular instances of DICOM data sets are encoded with UI VRs, which need to be globally unique. In this way, an image can be transferred across different devices. For example, the second device changes the image UID attribute because it deals with another instance of the same image. Each UID can be represented as:

UID:=<orgroot>.<suffix>

where, the <org root> part uniquely identifies an organization, with <suffix> being unique within the scope of the <org root>. The <org root> “1.2.840.10008” is reserved for DICOM-defined items (such as DICOM Transfer Syntaxes).

Any DICOM application on a given networked DICOM device is named as an *Application Entity (AE)*. One device can host multiple AEs, as in a case of an archive. AEs communicate between themselves by using the client/server model, in which clients are named as Service Class Users (SCUs) and the servers are named as Service Class Providers (SCPs). Each data exchange between SCU and SCP is called an *association*. The DICOM standard provides several different transport mechanisms for communication and data exchange between AEs. In more detail:

- DICOM Message Service (PS3.7) and Upper Layer Service (PS3.8);
- DICOM Web Service API and HTTP Service (PS3.18);
- Basic DICOM File Service (PS3.10);
- DICOM Real-Time Communication (PS3.22).

### 3.1. DICOM Message Service and Upper Layer Service

The DICOM Message Service and Upper Layer Service transport mechanism provide independence from the underlying network protocols. The Upper Layer Service allows communicating AEs to establish associations, exchange messages and terminate associations. It augments TCP/IP with some functions from the OSI upper layer protocols. The actual data exchange is done via service messages or commands, by using the *DICOM Message Service Elements (DIMSE)* services. In general, a DICOM AE uses two different ports: one for sending data (default port is 104), and another for receiving data.

Each DIMSE service usually has request/indication, and response/confirmation message components. The DIMSE provides two types of information transfer services: a notification service and an operation service. Notification services enable one DICOM AE to notify another about the occurrence of an event or change of state, while the operation services enable one DICOM AE to explicitly request an operation (e.g., storing an image) to be performed by another DICOM AE. As reported in Table 1, there are two groups of DIMSE services: DIMSE-C services applicable only to a Composite IOD and DIMSE-N services applicable only to a Normalized IOD.

DIMSE-C services are used for storage (C-STORE), query (C-FIND), retrieval (C-GET), and transfer (C-MOVE) of images, together with testing the link (C-ECHO). With one C-STORE-RQ can be sent only one image or one video clip which can be seen as IOD instance with multiple frames (e.g., ultrasound cine loops). DIMSE-N services are used for creation (N-CREATE), deletion (N-DELETE), updating (N-SET), and retrieval (N-GET) of some information, together with the action (N-ACTION), such as printing or initiating a storage commitment in terms like storage duration, retrieval latency and storage capacity, and notification of an event (N-EVENT-REPORT), such as printer status notifications or storage commitment confirmations [21].

**Figure 2:**
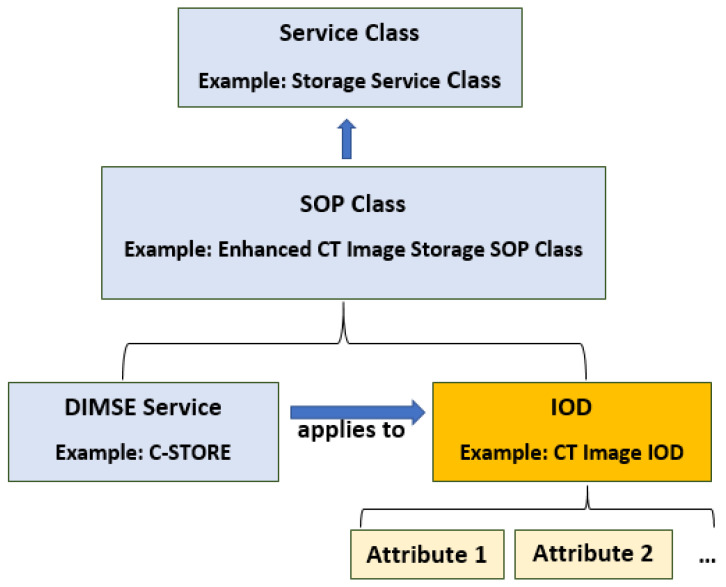
SOP Class Structure.

In DICOM, a service type is associated with the data (IODs) that it processes, and this is known as *Service-Object Pairs (SOPs)*. Each SOP belongs to some *SOP Class* (Figure 2), such as Enhanced CT Image Storage SOP Class or Digital X-Ray Image Storage SOP Class (PS3.4). One or more SOP Classes related to a specific function that needs to be accomplished by communicating AEs is a Service Class Specification (e.g., Storage Service Class or Verification Service Class). This specification plays a central role in defining DICOM conformance for each AE. DICOM defines two types of SOP Classes, Normalized and Composite. For DIMSE Services, Normalized SOP Classes are defined as the union of a Normalized IOD and a set of DIMSE-N Services, while Composite SOP Classes are defined as the union of a Composite IOD and a set of DIMSE-C Services.

#### 3.1.1. DICOM Messages

**Figure 3:**
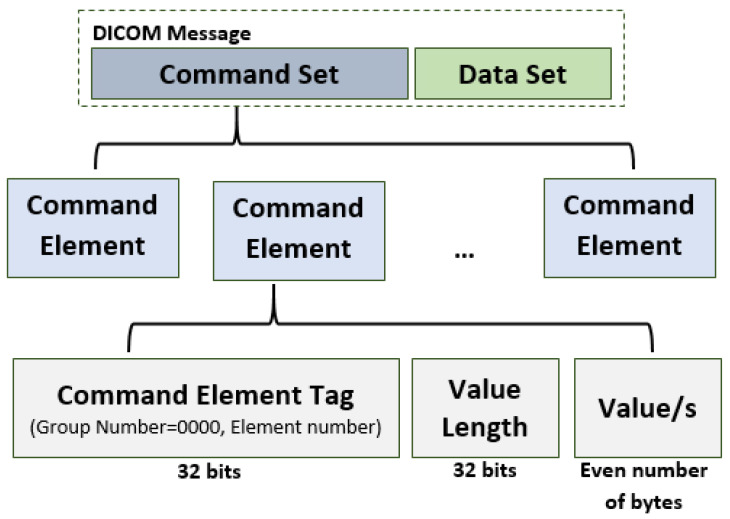
DICOM Message Structure.

Each DICOM message consists of two parts: *command set*, constructed from command elements, and data set. Figure 3 reports the general structure. In more detail, each command element consists of:

- Command Element Tag—an ordered pair of 16-bit unsigned integers (Group Number, Element Number). The Group Number for command element is always 0000;
- Value Length—a 32-bit unsigned integer representing the explicit length of the Value field in bytes;
- Value/s—an even number of bytes containing the Value(s) of the command element.

The command elements in a command set are ordered by increasing Command Element Tag number.

**Figure 4:**
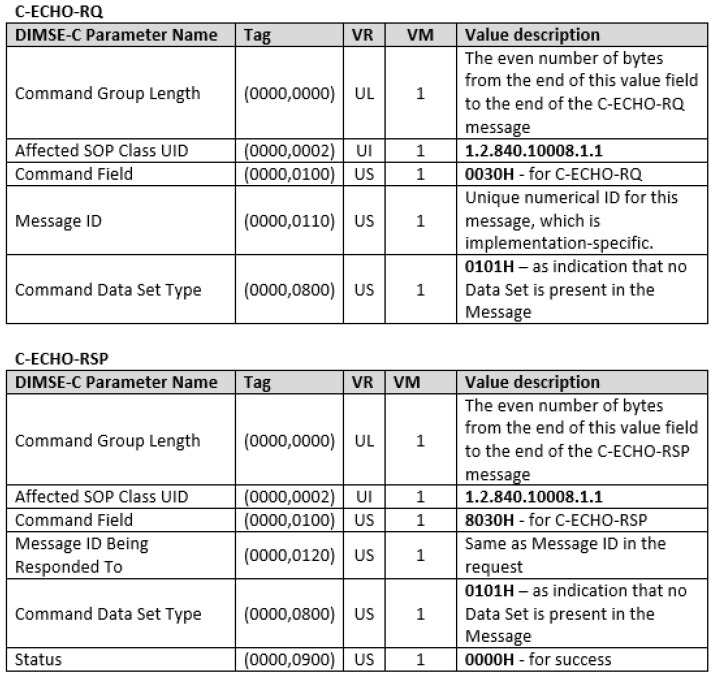
C-ECHO-RQ and C-ECHO-RSP Messages.

We can demonstrate how DICOM messages work with the simplest DIMSE service—C-ECHO service—by which one DICOM entity verifies that it is DICOM-connected with a peer DICOM entity. The SCU issues a C-ECHO request primitive (C-ECHO-RQ), for which an appropriate message (Figure 4) is constructed and sent to the SCP. Than the SCP issues a C-ECHO response primitive (C-ECHO-RSP), for which, an appropriate message is constructed (Figure 4) and sent back to the SCU. Each DIMSE service is identified by a predefined ID contained in the Affected Service Class UID value (e.g., 1.2.840.10008.1.1. for C-ECHO service). Each DICOM message type is identified by predefined ID contained in the Command Field value (e.g., 0030 for C-ECHO-RQ and 8030 for the C-ECHO-RSP). C-ECHO service is a part of the Verification SOP Class (without IOD data), which is a part of the Verification Service Class.

#### 3.1.2. Association Establishment and Termination

All DIMSE services are supported by the DICOM Upper Layer Services, such as: confirmed A-ASSOCIATE and A-RELEASE, or non-confirmed A-ABORT and P-DATA request services.

The first phase of communication between two DICOM compliant AEs is the association establishment. One AE initiates an association establishment to the other AE by using the A-ASSOCIATE request service. The two sides negotiate the application context (application service elements), the presentation context (how data will be presented), and the user information items (such as the maximum length of the application PDU, the service mod, maximum number of invoked or performed operations without confirmation for asynchronous mode, selection of the SCP/SCU role, specific options within a SOP Class). Default service mode is synchronous, where for each operation or notification, a response is needed, before invoking the next one. The asynchronous service mode allows AE to invoke several operations or notifications, without waiting a response, and in this mode, the order of responses can be mixed.

Operations and notifications can only be used on established association. They result in messages exchanged by using the P-DATA request service. For example, C-ECHO-RQ and C-ECHO-RSP are sent to the other site by using P-DATA request service on the established association. The C-MOVE service differs from the other services, because it triggers one or more C-STORE sub-operations on separate associations between the SCP and the third party AE.

A given association can be terminated in two ways: by orderly releasing from the AE that initiated the association (by using an A-RELEASE service) and by abrupt termination from any of the communicated parties (by using an A-ABORT service).

## 4. New Covert Channels in DICOM

In this section, we present several new network covert channels that are applicable to the DICOM standard and its DICOM Message Service and Upper Layer Service transport mechanism. As it will be detailed later, many different scenarios are possible: for instance, general-purpose covert communications, data exfiltration from compromised systems, or privacy leaking attacks targeting a specific individual.

### 4.1. System Model

The system model used for DICOM-based covert channels in this paper involves a covert sender (CS) and a cover receiver (CR). There are two different direct system submodels-DBasic and DPassive, both are applicable to all new direct covert channels presented in the following subsections.

**Figure 5:**
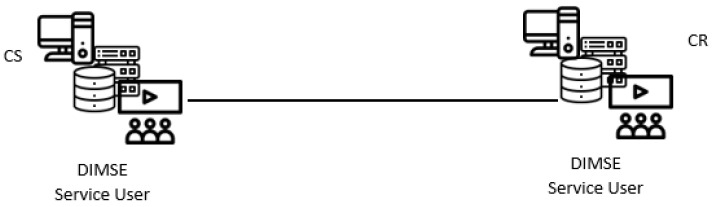
DBasic direct system submodel.

DBasic submodel involves normal DIMSE service, in which the AE that requests a given service (SCU) is the CS, while the AE that provide the service (SCP) is the CR (Figure 5). The CS hides the secret message into the normal DIMSE service traffic, by using exchanged DICOM messages. For example, the CS can be an installed malware application in the DICOM software of some modality, while the CR can be the doctor that views DICOM images on his/her workstation. Most often CS and CR are on the same intranet that belongs to some medical institution. But in a case of DICOM web access solutions, CS and CR can be located within different networks.

**Figure 6:**
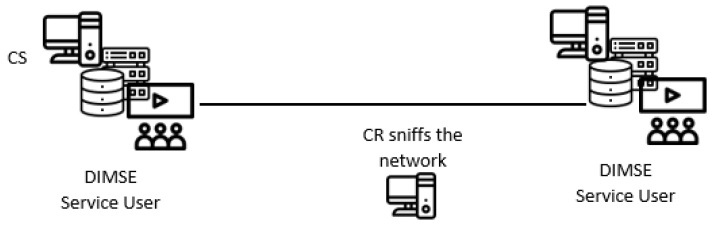
DPassive direct system submodel.

In the DPassive submodel, a CR is any device that passively analyzes the network traffic (see, Figure 6 for a sketch). The SCU AE or the SCP AE (as CS) can hide the secret message into the normal DIMSE service flow, while the CR can sniff the network traffic to intercept the DICOM messages, and extract the secret message. Concerning the scenario, the hidden communication attempt can be implemented within the hospital or medical facility.

**Figure 7:**
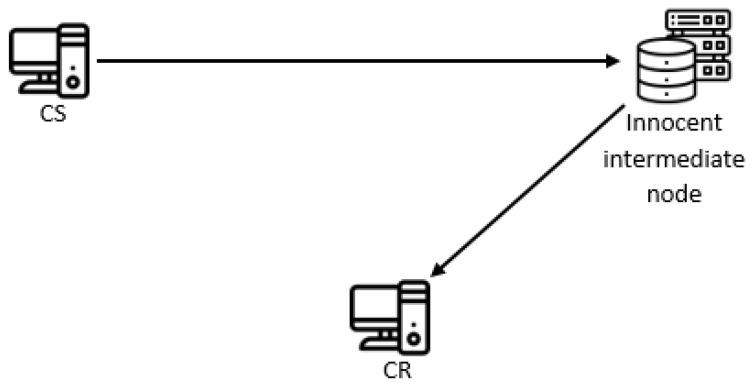
Indirect system model.

An indirect covert channel can be created based solely on the C-MOVE service, by which SCU asks an SCP (e.g., an archive) to send all Composite SOP instances that match the identifier to the third party AE (its AE Title is a value of the C-MOVE-RQ’s Move destination parameter). In this system model, C-MOVE SCP is an innocent intermediate node, the SCU is CS, while the third party AE is the CR (Figure 7). In reality, the third party AE is usually the SCU itself. For this to work, the intermediate node needs to know the IP address and the port of the third party AE, so it can map the given AE Title to them.

### 4.2. Covert Channels with Different Command Elements

There are several command elements that can be used for hiding data, and they comprise the first group of covert channels, denoted as CC1:

1. **Message ID**—The request messages for each DIMSE service contain a mandatory Message ID command element, which is unique within the scope of a given Association. Its tag is equal to (0000,0110), and it contains a randomly generated and implementation-specific numerical 16-bit value (its VR is US). All response messages contain a field **Message ID Being Responded To**, which should be the same with the Message ID value from the appropriate request message. So one can hide 16 bits in the value field of Message ID per a DIMSE request message.
  Since the Message ID field is randomized, the embedding and representation pattern are both “Random State/Value Modulation” (EN4.2 and RN4.2n) [10].
2. **Command Data Set Type**—The request or response messages for each DIMSE service contain a mandatory Command Data Set Type command element, with a tag equal to (0000,0800) and VR equal to US (with a fixed length of 2B). Its role is to indicate that a data set is present in the Message. It should be set to any value other than 0101H (Null), if there is a Data Set in the message (the previous versions of DICOM standard required this value to be set to 0102H). DIMSE services C-ECHO and N-DELETE do not have data set in their request and response messages. So one can hide 16 bits (different of Null) in the value field of Command Data Set Type per a DIMSE request message with a data set.
  The utilized field’s nature is almost entirely random (the value 0h cannot be used), so this covert channel is a form of the “State/Value Modulation” pattern (EN4 and RN4n), in particular it can be considered as a form of the sub-pattern “Random State/Value Modulation” (EN4.2 and RN4.2n) [10].
3. **Priority**—The request messages for the C-STORE, C-FIND, C-GET and C-MOVE operations contain a mandatory Priority command element, with a tag equal to (0000,0700) and VR equal to US (with a fixed length of 2B). Only three value are allowed: 0002H (Low), 0000H (Medium) and 0001H (High). So, one can create a 1-bit covert channel by using one of the values to represent a binary ‘1’, and other of the value to represent binary ‘0’.
  A semantically relevant field with a set of allowed values is modified which renders this covert channel as a variant of the “State/Value Modulation” pattern (EN4 and RN4n) [10].
4. **Attribute Identifier List**—The request messages for the N-GET operation contain an Attribute Identifier List command element, with the tag being equal to (0000,1005) and VR equal to AT. The value field contains an Attribute Tag for each of the *n* attributes applicable to this operation. So, one can create a 1-bit covert channel by using even values of *n* to represent a binary ‘1’, and odd values of *n* to represent a binary ‘0’.
  Again, this covert channel is a form of the “State/Value Modulation” pattern (EN4 and RN4n).
5. Some command elements have application-specific code values, like **Event Type ID** in N-EVENT-REPORT request, and **Action Type ID** in N-ACTION request, so they can be used for encoding hiding bits. All these values are 16 bits long (their VR is US). Their utilization would be a form of the “State/Value Modulation” (EN4 and RN4n), too.
6. Some command elements have textual values, like **Move Destination** in C-MOVE request and **Move Originator Application Entity Title** in C-STORE request which are AE, and **Error Comment** in some Status types of responses, which is LO. They can be used for hiding bits at the beginning and at the end of the string by using CC with whitespaces, cf. [5]. This channel is a form of the pattern EN3 “Elements/Features Enumeration” (EN3 and RN3n).

### 4.3. Covert Channel by Image Ordering in C-GET and C-MOVE Services

**Figure 8:**
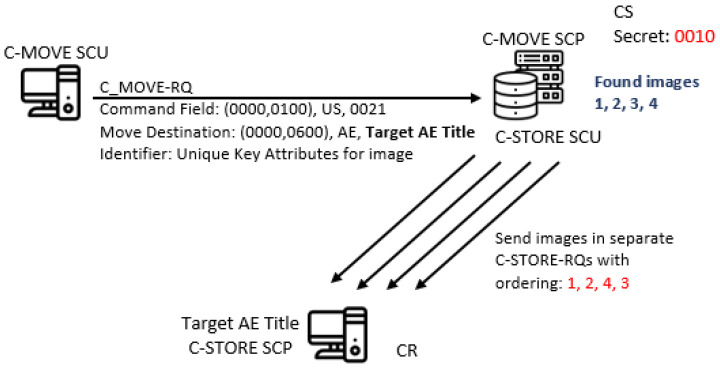
CC by image ordering in C-MOVE service.

The C-GET service fetches all Composite SOP instances that match a set of attributes given in the C-GET-RQ message’s data set (the set of attributes is called the Identifier parameter). It triggers one or more C-STORE sub-operations on the same association, for each matched instance. For example, one can send Patient ID as an attribute in the C-GET-RQ message’s data set, with a purpose to obtain all images for that specific patient. This will result in consecutive C-STORE-RQ messages for each separate found image, and their storing in the invoking AE. C-MOVE is similar to C-GET with one big difference, it can send (with C-STORE-RQ messages) all Composite SOP instances that match a set of attributes given in the C-MOVE-RQ message’s data set to any other AE (given as a value of **Move Destination** command element), see Figure 8. But usually the target AE is the same with the invoking AE.

The Identifier data set contains Query/Retrieve Level (0008,0052), which defines the level of the retrieval, and Unique Key Attributes, which may include Patient ID (0010,0020), Study Instance UIDs (0020,000D), Series Instance UIDs (0020,000E), and the SOP Instance UIDs (0008,0018).

Each image or each SOP instance has its own **Affected SOP Instance UID** with VR equal to UI. Each UI field is build from numerical characters and the dot character. One can introduce ordering for different UIs, for example, let $UI_{1}<UI_{2}$, if the number $ui_{1}$ obtained from $UI_{1}$ without dots is smaller than the number $ui_{2}$ obtained from $UI_{2}$ without dots, or another way, if the $UI_{1}$ has a lexicographical order smaller then $UI_{2}$.

So, one can hide $log_{2}n!$ bits in the order of the consecutive C-STORE-RQ messages for all found *n* images, by using their UIs for ordering in the specific pattern (CC2). For example, let UIs for four found images are 1, 2, 3 and 4 for simplicity, and if we choose the sequence 0010 to be represented with the pattern 1243, then sending this sequence corresponds to sending images in order 1, 2, 4 and 3. For this covert channel, the covert sender is the C-GET SCP (C-MOVE SCP), while the covert receiver is the C-GET SCU (other AEs specified by the C-MOVE SCU, see Figure 8). This covert channel is a form of the pattern EN2 (“Elements/Features Positioning”).

### 4.4. Covert Channel by Using Status Command Element in Responses

The responses for all DIMSE request messages contain a Status command element, with a value 0000H for success, and some other values, not always the same for all services. All DIMSE services, except C-FIND, support a status value 0210H for duplicate invocation, which indicates that the specified Message ID is allocated to another notification or operation.

One can create a unidirectional 1-bit covert channel (CC3) that belongs to the “State/Value Modulation” (ET4 and RT4n) pattern from the SCP to the SCU, per a response, by the following procedure:

- Sending a response message with Status value of 0210H after any DIMSE request message, except for C-FIND, can represent a binary ‘1’;
- Sending a response message with Status value different from 0210H (usually 0000H) after any DIMSE request message, except for C-FIND, can represent a binary ‘0’.

**Figure 9:**
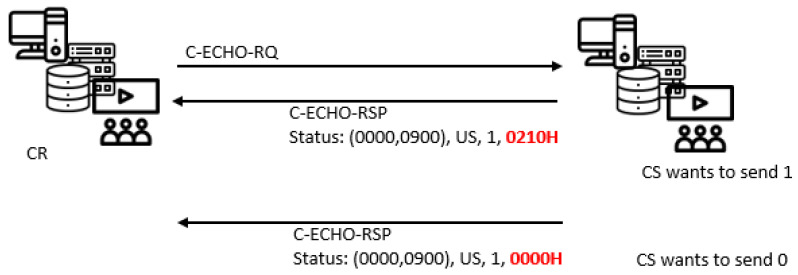
CC by using Status command element in C-ECHO service.

One example with the C-ECHO service is given in Figure 9.

### 4.5. Covert Channel by Using Cancellation Messages

The responses for C-FIND, C-MOVE and C-GET request messages can be cancelled by the invoking DIMSE Service User by issuing a C-CANCEL-FIND-RQ, C-CANCEL-MOVE-RQ or C-CANCEL-GET-RQ message, respectively.

One can create a unidirectional 1-bit covert channel (CC4) from the SCU to the SCP, per a request, by the following procedure:

- Sending a cancellation message immediately after C-FIND, C-MOVE or C-GET request messages can represent a binary ‘1’;
- Not sending a cancellation message immediately after C-FIND, C-MOVE or C-GET request messages can represent a binary ‘0’.

Due to the fact that a network packet (i.e., an element) does either occur or not, this covert channel belongs to the pattern ET2 “Event/Element Occurrence”.

### 4.6. Covert Channel by Using Message ID in C-MOVE Service

**Figure 10:**
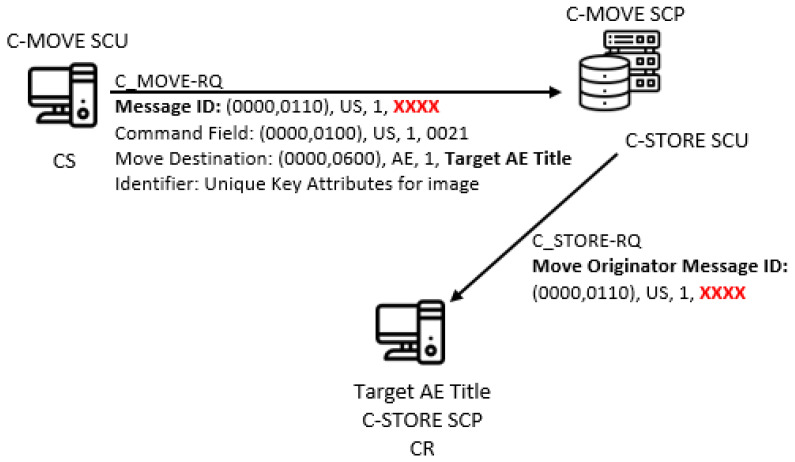
Indirect CC by using Message ID in C-MOVE Service.

Further, one can create an indirect CC by using the Message ID value in a C-MOVE-RQ message for hiding the secret bits (CC5). As stated above, this numerical 16-bit value is randomly generated, so by this CC one can hide 16 bits per a C-MOVE-RQ message. Random value fields are a subset of the “State/Value Modulation” pattern, namely EN4.2/RN4.2n (“Random State/Value Modulation”). The C-MOVE SCU requests from the innocent intermediate node with a role of C-MOVE SCP to send Composite SOP instances that match the attributes given in the Identifier data set to target AE. The identity of the target AE is given as a value of the Move Destination command element. For each Composite SOP instance, a separate C-STORE-RQ message is sent to the target AE. This CC is possible, because the C-MOVE SCP sends the Message ID value from the C-MOVE-RQ message, in each C-STORE-RQ message to the target AE, as a value of *Move Originator Message ID* command element (Figure 10).

Table 2 shows a summary of newly introduced covert channels.

## 5. Metrics and Countermeasures

### 5.1. Bandwidth Estimation

Without having a dataset with real traffic and statistics about the number of different DIMSE service messages exchanged during the working hours in typical hospitals, it is difficult to obtain a real bandwidth estimation of the proposed covert channels. Also, the average time for one DICOM modality scan/exam per a patient is not fixed and depends from the type of modality and on what part of the body it is being carried out. Table 3 reports data about an average duration of a specific scan/exam in the Department of Radiology and Biomedical Imaging at the University of California, San Francisco (https://radiology.ucsf.edu/patient-care/prepare, accessed on 20 December 2021).

So, if we use some modality as a covert sender, we will have between 0.5 and 4 DIMSE services per an hour in a worst case (in a case of one DIMSE service per scan/exam), which will lead to a bandwidth of at least 8 (in a case of 1 hidden bit per a service message) to 64 bits (in a case of 16 hidden bits per a service message) per an hour, depending of the used covert channel. If video is involved in the DICOM service or if the number of DIMSE services per scan/exam is greater than 1 (e.g., C-MOVE-RQ generate one or more consecutive C-STORE-RQ), the bandwidth will be greater than the estimation given previously, for the factor that is limited by the number of transmitted DIMSE messages per an hour.

The situation is different when the DIMSE services involve already stored DICOM files. One example is when the covert sender is a PACS archive, while the covert receiver is some workstation. In this case, if the CS produces a number of DIMSE messages per second denoted as *K* (*K* can be <1), the bandwidth will range from *K* to (16·K) bit/s, at least, depending of the used covert channel.

### 5.2. Robustness

The ability to protect the hidden message from interference introduced by third parties, or by the network, defines the robustness of the covert channel.

All the covert channels, except the CC4, are storage covert channels. A constant delay introduced by the network will not affect them. Even for the timing covert channel CC4, a constant delay will not be important, because the cancellation messages are carrying the Message ID of the proper request, so their receiving can be properly interpreted into the hidden bits. Their delay can only cause a non-cancellation of the request, but still the message will be received. For this channel, only packet losses, or more correct cancellation message losses, will have a negative impact on the hidden message receiving.

For CC2, the order in which images are sent to the covert receiver is important, and any variable delay which will cause reordering in image arrival times, will influence the robustness of the covert channel.

Variable network delay is not a problem for any covert channel in which the covert sender is the modality that needs first to generate a DICOM file, because the time intervals between consecutive scans/exams are much greater than any delay introduced by the network.

The robustness of the CC3 depends on the Message IDs that currently are in use in the SCP (covert sender) with all the AEs with which the SCP has a current communication. Thus, if the Message ID that belongs to the covert channel exists and SCP wants to send a binary ’0’, it can not do that because the specification expects the value 0210H to be sent.

The new covert channels are mainly robust against a third parties’ interference, because most of the DICOM traffic is happening in the hospital intranet. When one communication party is outside the hospital network, i.e. on the Internet, an adversary can perform a Man-in-the-Middle (MitM) attack and can influence the robustness of any covert channel by inserting, deleting or fabricating DIMSE messages.

### 5.3. Undetectability and Potential Countermeasures

The incapacity of third parties to distinguish covert traffic from a legitimate one is represented by the undetectability metric. The hospital security can apply different countermeasures for detection, limitation and/or prevention of DICOM covert channels in the form of passive and active wardens, traffic encryption or other measures against network sniffing, etc. The countermeasures for detection destroy or decrease the undetectability of any covert channel.

Covert channels CC1.1 and CC5 are difficult to detect because it is expected that each new DIMSE request message has a new random value in the Message ID. There is one exception—some implementations use a counter for the generation of Message ID and in this case, it is trivial to detect this covert channel. In any other case, one way to discover a possible covert communication is to look for the repetition of Message IDs in a short period, or at the number of occurrences of different 8-bit halves in Message ID (in case an unencrypted hidden message is transferred). The situation in which some halves occur several times, while other do not occur at all, or rarely, is a sign of a possible covert communication.

The same is true for CC1.2, i.e., the Command Data Set Type value. This CC can be prevented if the DICOM implementation uses only one value other than 0101H (Null) for signalling that there is a Data Set in the message. Thus, the previous versions of the DICOM standard that required this value to be set to 0102H were resistant to the CC1.2.

There are only three values for the Priority command element in C-STORE, C-FIND, C-GET and C-MOVE requests. If there is a recording about their average occurrences in the legitimate traffic, one can detect CC1.3 by counting their occurrences and look for significant deviations from their legitimate averages.

The detection of the CC1.4 is challenging because the number of attributes in the Attribute Identifier List command element is not predefined but user-defined, and there is no known legitimate parameter by which this number will be compared to.

Command elements that have application-specific textual values (used in CC1.5), provide some meaning for used applications, so anybody who is familiar with the application context, can easily detect their manipulation. In this way, a passive warden which complies to the application context can easily detect the covert channel CC1.5.

Covert channel CC1.6 can be prevented or detected by the measures suggested in [5].

Covert channel CC2 is also difficult to detect because the order in which the C-GET or C-MOVE SCP will send the Composite SOP instances that match the Identifier parameter is not predefined or known in advance. Thus, there is no known legitimate parameter by which this order can be compared to.

One way to detect the covert channel CC3 is to measure the number of occurrences of the value 0210H in the Status command element of the response after any DIMSE request message, except for C-FIND, and to compare it with the average number in the legitimate traffic samples. For hospitals, it is easy to obtain such a statistics from their networks, at any time.

Similarly, the covert channel CC4 can be detected by measuring the numbers of occurrences of C-CANCEL-FIND-RQ, C-CANCEL-MOVE-RQ or C-CANCEL-GET-RQ cancellation messages, and comparing them with their legitimate average occurrences.

## 6. Entropy-Based Detection Methodology for Real Scenarios and Known Covert Channels

According to Shannon [22], entropy is a measure of the uncertainty associated with a random variable. There are several papers that are using entropy to detect covert channels, such as those using information entropy [23] or relative entropy [24] for detection of network storage channels, corrected conditional entropy [25] for detection of network timing channels, etc. In this section, we will present a detection methodology of known covert channels based on the concept of cross-entropy, which is close to the concept of relative entropy given in [24]. Since we do not have access to real traffic, the testing of different parameters for this methodology is left for future work.

Given a discrete random variable *X*, with values $\left\{x_{1},\ldots ,x_{n}\right\}$, which have a probability distribution $P=\left(p\left(x_{1}\right),\ldots ,p\left(x_{1}\right)\right)=\left(p_{1},\ldots ,p_{n}\right)$, the entropy of *X* with *P* is formally defined as:

$$H\left(P\right)=-\sum_{i=1}^{n}p_{i}\log p_{i}.$$

Thus, in the real scenarios, the hospital can monitor their regular traffic, and can calculate its entropy separately for different properties, which are of interest. One example of the DICOM case is to calculate the entropy of a probability distribution of values for the command element Message ID, or its halves.

Cross-entropy between two probability distributions *P* and *Q* over the same underlying set *X* quantifies the difference between them:

$$H\left(P,Q\right)=-\sum_{i=1}^{n}p_{i}\log q_{i}.$$

When we have dataset with samples of covert communication *Q* that tries to mimic a probability distribution of regular communication *P*, the ideal case for the steganographic parties is that these two distributions are equal, which in the language of cross-entropy means that their cross-entropy H(P,Q) is equal to the entropy H(P) of *P*. By measuring how much the cross-entropy H(P,Q) differs from the entropy H(P) of regular traffic *P*, we can draw some conclusions about the covert communication. The actual difference between these two values can be seen as a measure of how a probability distribution *Q* is different from a reference probability distribution *P*, and is known as Kullback–Leibler divergence or relative entropy:

$$D_{KL}\left(P||Q\right)=H\left(P,Q\right)-H\left(P\right)$$

$$=-\sum_{i=1}^{n}p_{i}\log q_{i}+\sum_{i=1}^{n}p_{i}\log p_{i}$$

$$=-\sum_{i=1}^{n}p_{i}\log\left(p_{i}/q_{i}\right)$$

The hypothesis is that if the adversary is using non-encrypted text as secret message, the produced covert probability distribution *Q* will differ a lot from the *P* of regular traffic. Here we will define the detecting methodology for a general case. Let the adversary use non-encrypted text with characters of the length *N* bits (for example, for ASCII characters, *N* is 7 or 8). The passive warden can use the following steps:

- *Step 1.* Choose the property which the warden wants to protect and which is potentially used by the covert channel (e.g., Message ID, Command Data Set Type, Status, Priority) and choose the network flow that it wants to examine.
- *Step 2.* Perform the following:
  – For a CC where the hidden bits are encoded directly into the values (e.g., Message ID, Command Data Set Type), take the consecutive property values from the suspected network flow, and from them construct the array of consecutive *N*-bit words. Calculate the probability distribution *Q* of elements in this array.
  – For a k-bit CC where *k*-bit values correspond to $2^{k}$ different states (e.g., Priority, Status), take the property’s events that correspond to $2^{k}$ different states and assign proper *k*-bit values (binary ‘1’ and ‘0’ for 1-bit CC) from the suspected traffic form the string of appropriate *k*-bit values and divide the string into an array of consecutive *N*-bit words. Calculate the probability distribution *Q* of elements in this array.
- *Step 3.* Perform the same procedure but for the pre-recorded regular traffic, and calculate the probability distribution *P* of elements in the obtained array. Calculate the entropy H(P) of *P*.
- *Step 4.* Calculate the cross-entropy H(P,Q), and compare it with the entropy H(P) of *P*. If there is a significant difference, the suspected network flow is probably used for clandestine communication.

In the future work, above-mentioned approach must be tested in real scenarios, and a significant difference, together with other parameters of the methodology (e.g., number of needed samples, or time interval for suspected flow) needs to be defined. However, we will perform an experimental evaluation under testbed conditions in the remainder.

### Experimental Evaluation

**Figure 11:**
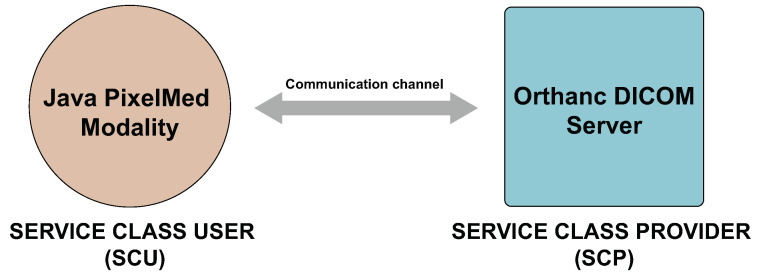
Experimental scenario.

**Figure 12:**
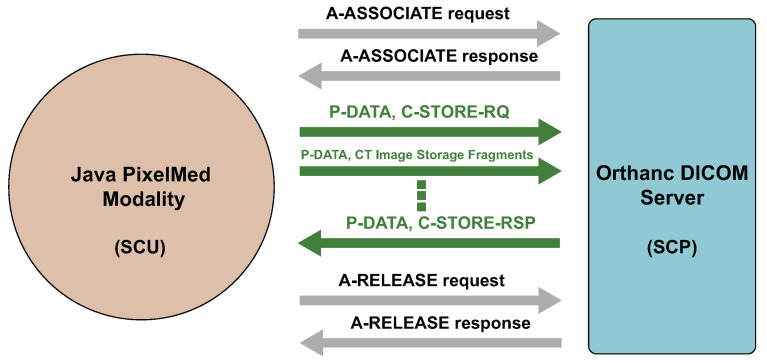
Detailed communication between SCU and SCP.

Due to the lack of datasets that we can use for our research for the evaluation of the covert channels, we created an experimental scenario. It consists of a modality (as a SCU) that sends images to a DICOM server (as a SCP), see Figure 11. Detailed communication between the SCU and SCP can be seen in Figure 12. For this purpose, we used the Elastic Compute Cloud (https://aws.amazon.com/ec2/, accessed on 5 December 2021) (EC2) service from the Amazon Web Services (AWS).

We created two EC2 instances. In our scenario one of the instances plays the role of a modality, while the other is a DICOM server. The modality instance is from the type t2.medium, with the following configuration: AMD64 architecture, 2 vCPU, 4 GB RAM and 30 GB General Purpose SSD (gp2) storage. The server instance is from the type t2.large, with AMD64 architecture, 2 vCPU, 8 GB RAM and 30 GB SSD gp2 storage. Both instances run the Microsoft Windows Server 2019 Base 64-bit operating system with the code name “ami-0b17e49efb8d755c3”.

We implemented the modality in the Java programming language using the PixelMed library (http://www.pixelmed.com/dicomtoolkit.html, accessed on 20 December 2021). It is a library which provides implementations of functions that can be used for creating associations between SCUs and SCPs, sending DICOM files, receiving, moving and other DICOM operations.

For our experiments we used the Orthanc DICOM server (https://www.orthanc-server.com/, accessed on 20 December 2021) version 1.9.7. It is an open source and lightweight server with an ubiquitous web interface. Orthanc has support for all DICOM functions, REST API to automate the flow of images and SDK for integration with native applications.

For our experimental evaluation, we also recorded the traffic on the DICOM server. For this purpose, we used the tool Wireshark version 3.6.0 (https://www.wireshark.org/, accessed on 20 December 2021).

We created three variants of the experimental scenario:

- Scenario with legitimate traffic
- Scenario with implemented covert channel—sending an ASCII covert message
- Scenario with implemented covert channel—sending an AES encrypted covert message

In the first variant, the modality sends DICOM images to the Orthanc server. For our experimental scenario, in all variants we used images from the Public Lung Database (https://www.via.cornell.edu/databases/simbadb.html, accessed on 20 December 2021) [26]. It contains Computed Tomography (CT) images showing large lesions in the lungs. This dataset also consists of images from different categories such as: Emphysema, Single Large Nodules, Single Small Nodules, Repeat Single Session, CT Angiography, and others. For our experiments we used the Emphysema subset. It contains more than 5000 DICOM images. We used the images located in the following folder hierarchy EM0085/1.2.826.0.1.3680043.2.656.4.1.7.83/S02A01. Due to the protection of the patients’ privacy, this dataset has been anonymized. In this variant of our experiments, the modality takes a picture from the folder and sends it to the Orthanc server. The images are sent in one association and a random Message ID is generated for each DICOM message.

In the second variant of our experimental scenario, we implemented the covert channel with Message ID (CC1.1). The modality as a covert sender sends ASCII covert message to the DICOM server as a covert receiver. With one DICOM message, 2 hidden ASCII characters are sent.

In the third variant, we used the same implementation of the covert channel CC1.1 from the variant 2, but here an AES encrypted covert message is sent.

In all variants, the sending of DICOM messages is conducted in a random time interval of about 15 min, which means that for a time interval of 1 h about 4 images are sent. We recorded the traffic on the server side for a time period of 12 and a half hours at most. In this way, we are using 50 DIMSE STORE-RQ services for storing 50 images in the archive per each variant. For the covert traffic this corresponds to the sending of 100 characters.

To show some insights of how it is likely to capture irregularities in Message ID half values, we are using the same number of DIMSE STORE services for three types of traffic: regular traffic, covert traffic with ASCII characters and covert traffic encrypted with AES block cipher.

**Figure 13:**
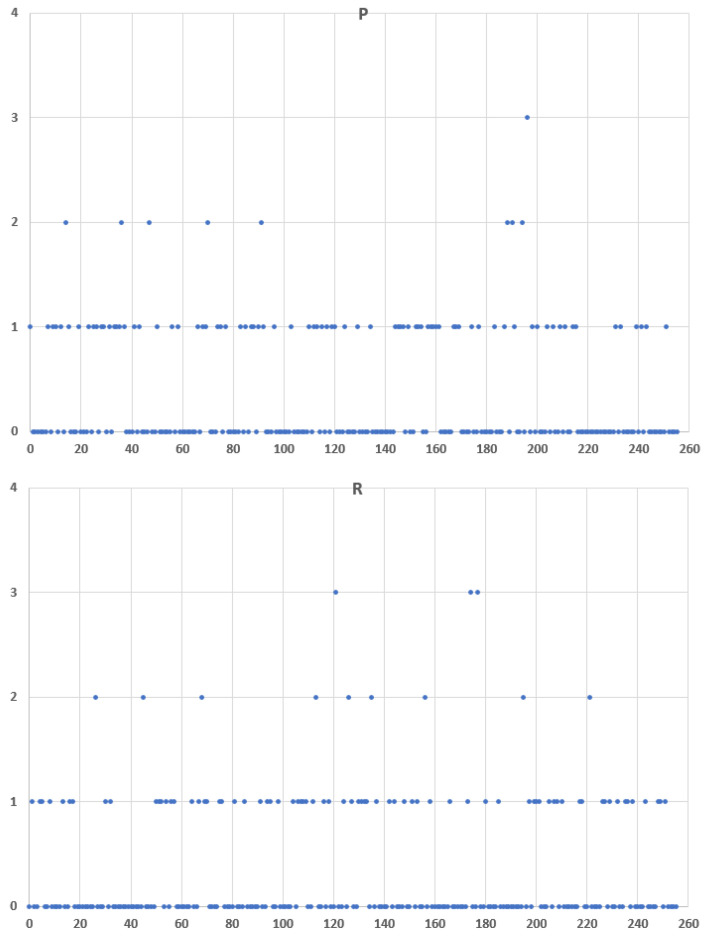
Message ID half value frequency distributions for regular traffic for two datasets, *P* and *R*, where x: Message ID half value and y: frequency.

Figure 13 plots the frequency distribution of the 8-bit Message ID half values for regular traffic for two datasets, *P* and *R*. One can notice that there are quite similar.

**Figure 14:**
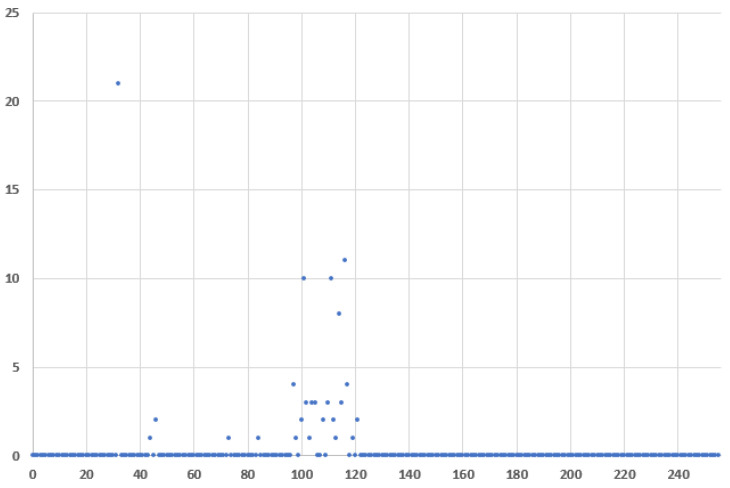
Message ID value frequency distributions for covert traffic with ASCII characters, where x: Message ID half value and y: frequency.

**Figure 15:**
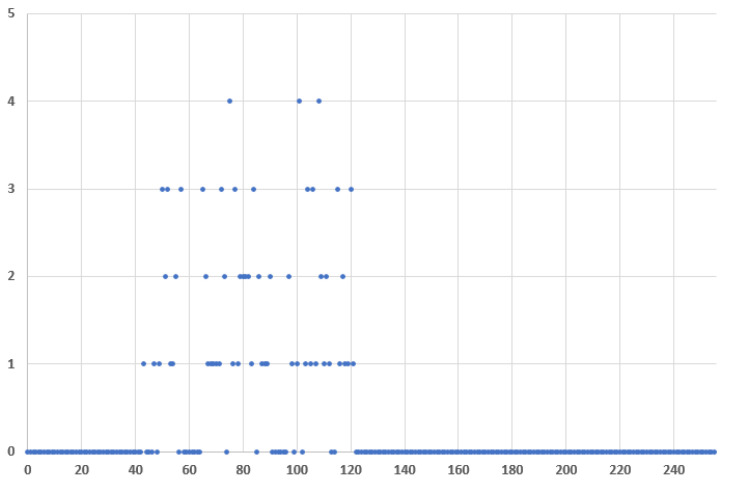
Message ID value frequency distributions for covert traffic encrypted with AES, where x: Message ID half value and y: frequency.

Figure 14 and Figure 15 plot the frequency distribution of the 8-bit Message ID half values for covert traffic with ASCII characters and encrypted with AES block cipher, respectfully. The difference in the first case from the regular traffic is more obvious, than in the second case when AES is used.

Because we do not have real hospital traffic for a long period of time, we can use the probability distributions for the obtained datasets, *P* and *R* for regular traffic datasets, $Q_{1}$ for covert traffic dataset with ASCII characters and $Q_{2}$ for covert traffic dataset encrypted with AES.

From the Table 4, one can notice that cross-entropy between *P* and *R* of two regular traffic datasets is pretty close to the cross-entropy between *P* and $Q_{2}$ (between the first regular traffic dataset and the covert traffic dataset encrypted with AES). Also, both of them are quite different from the cross-entropy between *P* and $Q_{1}$ (between the first regular traffic dataset and the covert traffic dataset with ASCII characters). So, even for small datasets and beside the existence of big differences between the entropy H(P) and calculated cross-entropy values between the *P* and other datasets, one can conclude that another regular traffic dataset *R* and covert traffic dataset encrypted with AES $Q_{2}$ have almost the same distance to H(P), while the covert traffic dataset with ASCII characters $Q_{1}$ shows a much larger distance to H(P). This means that covert traffic with ASCII characters can easily be distinguished from regular traffic, even for smaller samples. For the AES encrypted covert traffic this is not a case. Table 5 confirms this behaviour: it compares the obtained cross-entropy values between the second regular dataset *R* and other used datasets.

We will leave the testing of different parameters of the methodology (e.g., threshold value for detecting a significant difference between the cross-entropy H(P,Q) and the entropy H(P) of regular traffic) for future work and will conduct these experiments when real hospital traffic will become available to us.

## 7. Conclusions

In this paper, the DICOM Message Service and Upper Layer Service transport mechanism was analyzed regarding its susceptibility to network steganographic techniques. Ten new network covert channels were described, nine covert channels are direct covert channels and one is an indirect covert channel. Three basic network steganographic parameters (bandwidth, undetectibility and robustness) were discussed and evaluated for the proposed covert channels. Several potential countermeasures were suggested which can help in building a warden that can improve the DICOM security. In addition, we implemented and evaluated an entropy-based detection heuristic using a testbed. For the experimental evaluation, we implemented a covert channel that modulates the Message ID (CC1.1), where the covert sender is a DICOM client and the covert receiver is the DICOM archive, and we generated three datasets, one with legitimate traffic and two with covert traffic (secret data in clear ASCII form and in AES encrypted form). As demonstrated, the detectability of our channels can be considered challenging, especially when secret data is encrypted with a strong cipher. In future work, we plan to investigate further detection and elimination methods for DICOM-based covert channels using real-world traffic.

## Figures and Tables

**Table 1:** DIMSE Services.

Name	Group	Type	Description
C-STORE	DIMSE-C	operation	It allows SCU to send a Composite SOP instance to an SCP, for SCP to store it
C-GET	DIMSE-C	operation	It allows SCU to fetch one or more Composite SOP instances from an SCP, based upon the attributes supplied by the SCU
C-MOVE	DIMSE-C	operation	It allows SCU to move one or more Composite SOP instances from an SCP, to a third party AE (often SCU itself), based upon the attributes supplied by the SCU
C-FIND	DIMSE-C	operation	It allows SCU to send attribute string that is matched against the attributes of SOP instances managed by the SCP. SCP returns for each match a list of requested attributes and their values.
C-ECHO	DIMSE-C	operation	It allows SCU to verify end-to-end communications with an SCP
N-EVENT-REPORT	DIMSE-N	notification	It allows SCU to report an event about a SOP instance to an SCP
N-GET	DIMSE-N	operation	It allows SCU to request the retrieval of information from an SCP
N-SET	DIMSE-N	operation	It allows SCU to request the modification of information by an SCP
N-ACTION	DIMSE-N	operation	It allows SCU to request an SCP to perform an action
N-CREATE	DIMSE-N	operation	It allows SCU to request an SCP to create an instance of a SOP Class
N-DELETE	DIMSE-N	operation	It allows SCU to request an SCP to delete an instance of a SOP Class

**Table 2:** Summary of the introduced covert channels.

CC	System Sub−Model	Embedding Pattern	Representation Pattern	Bits per Service Message	DIMSE Services	Message Type
CC1.1	DBasic, DPassive	EN4.2	RN4.2n	16	All	Request
CC1.2	DBasic, DPassive	EN4.2	RN4.2n	16	All	Request\response
CC1.3	DBasic, DPassive	EN4	RN4n	1	C-STORE, C-FIND, C-GET, C-MOVE	Request
CC1.4	DBasic, DPassive	EN4	RN4n	1	N-GET	Request
CC1.5	DBasic, DPassive	EN4	RN4n	16	N-EVENT-REPORT, N-ACTION	Request
CC1.6	DBasic, DPassive	EN3	RN3n	1	C-MOVE, C-STORE or All	Request or response
CC2	DBasic, DPassive	EN2	RN2n	$log_{2}n!$	C-GET, C-MOVE with consecutive C-STORE	Request
CC3	DBasic, DPassive	EN4	RN4n	1	All without C-FIND	Response
CC4	DBasic, DPassive	ET2	RT2n	1	C-FIND, C-MOVE and C-GET	Cancellation message request
CC5	Indirect	EN4.2	RN4.2n	16	C-MOVE with consecutive C-STORE	Request followed by response

**Table 3:** Average duration of one scan/exam in the Department of Radiology and Biomedical Imaging at the University of California, San Francisco.

Modality Scan/Exam	Average Duration	Average No. of Scans per Hour
Diagnostic X-ray exam	15 min	4
MRI scan	45–60 min per body part	1.33–1
CT scan	15 min	4
PET/CT scan	120 min	0.5
Ultrasound exam	30–60 min	2–1
US Hysterosonogram exam	45–60 min	1.33–1
Arthrogram exam	45 min	1.33
Myelogram exam	60 min	1
Discogram exam	120 min	0.5

**Table 4:** H(P) for the first regular traffic dataset and obtained cross-entropy values with other datasets.

H(P)	H(P,R)	$H(P,Q_{1})$	$H(P,Q_{2})$
1.9375	0.4214	0.1214	0.4059

**Table 5:** H(R) for the second regular traffic dataset and obtained cross-entropy values with other datasets.

H(R)	H(R,P)	$H(R,Q_{1})$	$H(R,Q_{2})$
1.9029	0.5340	0.1765	0.4551

## Abbreviations

ACR	American College of Radiology
AE	Application Entity
AES	Advanced Encryption Standard
AT	Attribute Tag
CC	Covert Channel
CR	Covert Receiver
CS	Covert Sender
CT	Computed Tomography
DICOM	Digital Imaging and COmmunication in Medicine
DIMSE	DICOM Message Service Elements
DIMSE-C	DIMSE Composite
DIMSE-N	DIMSE Normalized
EC2	Elastic Compute Cloud
GAN	Generative Adversarial Networks
HIS	Hospital Information System
HL7	Health Level Seven
ID	Identifier
IODs	Information Object Definitions
LO	Long String
LSB	Least Significant Bit
MR	Magnetic Resonance
MRI	Magnetic Resonance Imaging
NEMA	National Electrical Manufacturers Association
PACS	Picture Archiving and Communication Systems
PET	Positron Emission Tomography
RIS	Radiology Information System
RQ	Request
RSP	Response
SCPs	Service Class Providers
SCUs	Service Class Users
SOPs	Service-Object Pairs
UI VR	Unique Identifier Value Representation
UID	Unique Identifier
UL	Unsigned Long
US	Unsigned Short
VM	Value Multiplicity
VR	Value Representation
